# Mistrust and Beliefs in Conspiracy Theories Differently Mediate the Effects of Psychological Factors on Propensity for COVID-19 Vaccine

**DOI:** 10.3389/fpsyg.2021.683684

**Published:** 2021-07-07

**Authors:** Luca Simione, Monia Vagni, Camilla Gnagnarella, Giuseppe Bersani, Daniela Pajardi

**Affiliations:** ^1^Institute of Cognitive Sciences and Technologies, CNR, Rome, Italy; ^2^Department of Humanities, University of Urbino, Urbino, Italy; ^3^Department of Neurology and Psychiatry, Sapienza, University of Rome, Rome, Italy; ^4^Department of Medico-Surgical Sciences and Biotecnologies, Sapienza, University of Rome, Rome, Italy

**Keywords:** vaccine propensity, vaccine hesitancy, conspiracy theories, mistrust, paranoia, death anxiety, COVID-19

## Abstract

Vaccination is considered a key factor in the sanitary resolution of the COVID-19 pandemic. However, vaccine hesitancy can undermine its diffusion with severe consequences on global health. While beliefs in conspiracy theories, mistrust in science and in policymakers, and mistrust in official information channels may also increment vaccine hesitancy, understanding their psychological causes could improve our capacity to respond to the pandemic. Thus, we designed a cross-sectional study with the aim of probing vaccine propensity in the Italian population and explored its relationship with sociodemographic and psychological variables, and with misbeliefs in COVID-19. A battery of questionnaires was administered to a sample of 374 Italian adults during the first national lockdown (April 2020). The materials included an original instrument—Beliefs in COVID-19 Inventory—and questionnaires measuring perceived stress, anxiety, death anxiety, psychological distress, psychoticism, paranoia, anger, and somatization. The exploratory factor analysis (EFA) on Beliefs in COVID-19 suggested the existence of three factors: belief in conspiracy theories, mistrust in medical information, and mistrust in medicine and science. These factors were positively correlated with female sex, age, religious beliefs, psychiatric conditions, and psychological variables, while negatively correlated with education levels. We conducted a mediation analysis by means of a structural equation model, including psychological factors as predictors, beliefs in COVID-19 scales as mediators, and vaccine propensity as an outcome. The model showed that death anxiety had a direct positive effect on the propensity to get vaccinated. It also showed that death anxiety reduced the propensity to get vaccinated through a mediated path in believing in conspiracy theories, whereas paranoia was linked to a reduction in vaccination adherence with the mediation effect of mistrust in medical science. Psychological distress reduced vaccination propensity by increasing both conspiracy beliefs and mistrust. On the other hand, anxiety increased the propensity to get vaccinated through a decrease in both belief in conspiracy theories and mistrust in science. Our results suggest that psychological dimensions are differentially related to belief in conspiracy theories, to mistrust in science, and to the propensity to get vaccinated. Based on this result, we propose an original interpretation of how conspiracy beliefs build on a paranoid and suspicious attitude. We also discuss the possible clinical implications of treatment for such pathological beliefs.

## Introduction

Given the extent and severity of COVID-19 around the world, global population vaccination has been proposed as the key to halting the spread of the pandemic. As early as last March-April 2020, science began to find vaccine remedies for COVID-19 (Lurie et al., 2020) and governments supported the academic community and pharmaceutical industry in identifying a safe and effective vaccine remedy (Kaur and Gupta, 2020). Vaccines would be suitable and necessary to reduce transmission, hospitalization, and the high number of intensive care patients. However, global vaccination is not easy to reach as a goal: It requires not only a sufficient health system capacity, but also efficacy strategies capable of bringing people to accept and trust in the vaccine and in those who deliver it. Some studies already highlighted in the early stages of the pandemic that it was urgent to plan actions to reassure the general population and to promote trust in biomedical research (Palamenghi et al., 2020). In fact, the scientific evidence relating to COVID-19 has been found to be so uncertain and contradictory that it has changed, even in the medium term, the social representation of scientific knowledge (Provenzi and Barello, 2020). For all these reasons, a skeptic position toward vaccination may emerge in the population. Vaccine hesitancy is a well-known phenomenon indicated by the WHO as one of the main global health threats (Sallam et al., 2021).

Vaccine hesitancy can be defined as the delay in acceptance or refusal or reluctance of vaccine acceptance despite the availability of vaccination services (McDonald, 2015; Mannan and Farhana, 2020; Sallam et al., 2021). Hesitancy behavior should be understood as a continuum between those who totally accept and those who totally refuse all vaccines, with the hesitant individuals placed in between these two extremes (Sato, 2018). Previous studies have shown that some sociodemographic factors are associated with vaccine hesitation or refusal. In particular, females tend to be hesitant and more skeptical of the COVID-19 vaccine (Lazarus et al., 2020; Mannan and Farhana, 2020; Lin et al., 2021; Murphy et al., 2021; Patelarou et al., 2021). Also, younger age, higher levels of education, and religious beliefs have been related to vaccine hesitancy and resistance (Malik et al., 2020, Palamenghi et al., 2020; Lin et al., 2021; Murphy et al., 2021). However, some studies reported higher vaccine acceptance among females (Al-Mohaithef and Padhi, 2020; Lazarus et al., 2020) and those with higher education levels (e.g., Lazarus et al., 2020).

### Psychological Factors Associated With Vaccination Behavior

Trust in vaccination seems to be affected also by psychological factors, such as anxiety or perceived stress. The psychological condition of the population was greatly affected by the pandemic and the related restrictive measures. Distress, depression, and death anxiety characterized the psychological response to the first pandemic spread (e.g., Simione and Gnagnarella, 2020) and the prevalence of psychological symptoms greatly increased during this period (Ran et al., 2020). In addition, hostility and anger increased as a response to quarantine and lockdown measures (Duan et al., 2020). In this weak psychological condition, the feelings of fear of dying, anguish, vulnerability, and insecurity that the person can experience could lead to higher levels of confidence in COVID-19 vaccination (Kang and Jung, 2020; Mannan and Farhana, 2020; Patelarou et al., 2021). However, anger and negative emotions were also related to lower levels of vaccine acceptance (Betsch and Böhm, 2016; Sun et al., 2021). Thus, differential psychological symptoms, ranging from stress to death anxiety, seems to be associated with either higher or lower vaccine acceptance. Chou and Budenz (2020) proposed that key factors in determining the influence of emotions and psychological factors on COVID-19 vaccine propensity are conspiracy beliefs, mistrust or skepticism, and misinformation. Considering these factors would lead to a better comprehension of the mechanisms by which certain psychological variables increase the vaccine hesitancy, while others reduce it.

### Trust in Medicine and Science Affects Vaccine Propensity

The issue of trust in medicine and science associated with vaccination has been the subject of many studies over the years even before the COVID-19 pandemic. Studies on trust in vaccination and medicine generally focused on a single element or phenomenon more than a generalized trust (Larson et al., 2018). Instead, trust is a multilayered concept (Chryssochoidis et al., 2009), which includes sociocultural and personality factors, but also the perceptions of institutions, the health system, the capacity of science and pharmaceutical companies, and the reliability of the health professionals involved (Liu and Yang, 2020; Patelarou et al., 2021). As highlighted by some authors (Larson et al., 2018; Liu and Yang, 2020), in the context of vaccination, trust may be considered on three levels: trust in the product (e.g., hepatitis B vaccine), trust in the vaccine provider (e.g., healthcare professionals), and trust in policymakers (e.g., the government and the healthcare system). Each level of trust may influence the public's safety and effectiveness perception of the vaccine, and consequently the adherence to vaccination campaigns. COVID-19 vaccine acceptance seems to be influenced by variables common to those recorded for other vaccines (efficacy, minor adverse effects, and protection duration; Kreps et al., 2020) or in previous health emergencies, such as HIV, SARS, MERS, and Ebola (Lazarus et al., 2021). However, the COVID-19 scenario was also characterized by specific factors that could increase mistrust in science and health experts, such as the unusually rapid speed of vaccine development, the uncertainty about medical information, and the documented concerns about vaccine safety (see Chou and Budenz, 2020). These specific factors could undermine people's trust in institutional actors, and then influence their willingness to engage in preventive health behaviors. Thus, both trust in medical science and trust in policymakers seem to be important factors in determining vaccine adherence.

### Beliefs in Conspiracy Theories Decrease Vaccine Propensity

Associated with distrust in science and skepticism, the literature highlights the prominent role of conspiracy theory beliefs. Conspiracy theories can be defined as a “subset of false beliefs in which the ultimate cause of an event is believed to be due to a plot by multiple actors working together with a clear goal in mind, often unlawfully and in secret” (Swami and Furnham, 2014, p. 220). One of the central aspects of the conspiracy beliefs comes from distrust in political institutions, which can also lead to resistance to important medical and public health interventions (Ford et al., 2013; Oliver and Wood, 2014; Landrum and Olshansky, 2019), without diminishing the seriousness of the pandemic threat. In fact, according to Hornsey et al. (2021), conspiratorial people tend to feel alienated, mistrustful, and angry. Moreover, they tend to be predominantly focused on their own personal interest and well-being, and less concerned with the well-being of those close to them. Conspiratorial thinking seems to protect these people from the anxiety and anguish of death, leading them to deny the problem of COVID-19 infection and, therefore, also to refuse the vaccine.

Vaccine distrust of conspiratorial people could be linked to a generic belief system characterized by negative attitudes toward powerful groups, such as medical or political institutions (Bertin et al., 2020). Distrust and suspicion in conspiracy theorists are also accentuated in the present pandemic scenario by the economic interests of pharmaceutical companies related to the vaccine, where Big Pharma may exaggerate the benefits of vaccines by minimizing their dangers (Jolley and Douglas, 2014; Hornsey et al., 2021).

### Role of Trust in Information Sources in Vaccination Behavior

Another important factor in determining vaccine hesitancy is the information relative to COVID-19 and to its vaccines (Sherman et al., 2020). This is particularly true for the present situation where we only have first-generation vaccines whose long-term effects are unknown. In turn, this lack of information could lead to both vaccine hesitancy and distrust in the institutional organizations providing the vaccine. The source of information has also an effect on trust in science and vaccine hesitancy. Malik et al. (2020) found that participants who had more trust in medicine got information from healthcare workers and health officials, whereas those who collected sources from social media had less trust in medical science. Similar results were found by Patelarou et al. (2021), showing that those who got information from newspapers, television, radio, and government agencies had more trust in the COVID-19 vaccine than those who had self-perceived knowledge or collected information through social or online media. In fact, misinformation is more available on the internet where the information may be less accurate or verified (Liu and Yang, 2020; Obiała et al., 2020; Patelarou et al., 2021). Skeptics also use online platforms to advocate vaccine refusal. Hussain et al. (2018) found that as many as 50% of tweets about vaccination contain anti-vaccine beliefs, and this may increase the perception of vaccination risks and decrease perceptions of the risks of non-vaccination (Benecke and DeYoung, 2019).

Lastly, information plausibility and processing impact the formation of vaccine-related behaviors. Mannan and Farhana (2020) highlighted that many government decisions may be unwelcome as they are felt to be disproportional with the pandemic status or not justified enough by scientific knowledge about COVID-19. This could undermine trust in governments and in scientific national committees who issue lockdown or restrictive measures to prevent the virus spreading, and in turn this may affect vaccine propensity, as vaccines would be offered to the population by the very same actors.

Overall, these studies point out the important role of information in determining vaccine propensity. In particular, the trust in the information source, the understandability of information, and how it is received by individuals are all important determinants of the effect of information on vaccine hesitancy (Pagliaro et al., 2021).

### Mistrust, Misinformation, and Conspiracy Beliefs Could Mediate the Effect of Psychological Factors on Vaccine Propensity

Importantly, mistrust and belief in conspiracy theories are related to psychological factors. Several studies have indicated the role played by distress in driving people to adhere to conspiracy theories as a strategy to find meaning, order, or controllability of otherwise ambiguous events (Swami et al., 2017; Georgiou et al., 2020). van Prooijen and Douglas (2018) found that a higher degree of conspiracy beliefs arises from hypervigilance and reactions to stressful situations. Conspiracy theories beliefs seem also to be strongly associated with underlying psychopathological traits, which make a person more likely to develop erroneous beliefs (Hart and Graether, 2018; Georgiou et al., 2019, 2020). For example, significant correlations with schizotypy (Buchy et al., 2007; Barron et al., 2014, 2018; Eisenacher and Zink, 2017) and paranoia (Murphy et al., 2021) were found. In particular, paranoid ideation seems to be associated with mistrust, suspicion, and conspiracy beliefs (Imhoff and Lamberty, 2018). The existential threat could also fuel the belief in conspiracy theories, in particular when an antagonistic outgroup can be identified, e.g., Big Pharma, healthcare workers, or policy makers (van Prooijen, 2020). Conspiracy beliefs, mistrust, and misinformation are related to both decreased vaccine acceptance and worst psychological conditions. Following Chou and Budenz (2020), these factors could potentially mediate the effect of psychological state on vaccine acceptance, by increasing fear of vaccination and then hesitancy.

### Aim and Hypotheses of the Present Study

In the present study, we investigated this complex relationship pattern between psychological factors, mistrust, and COVID-19 vaccine propensity during the first stage of the pandemic, when the vaccines were not yet available. To this aim, we administered an online battery of questionnaires to a general sample of the Italian population. In this battery, we collected data about sociodemographic information that were relevant for vaccination behavior or psychological well-being, i.e., sex (Malik et al., 2020), education (Lazarus et al., 2020), religious beliefs, familiar, and economic status (Murphy et al., 2021), working condition, i.e., if in smart working (Mari et al., 2021) or in the healthcare system (Simione and Gnagnarella, 2020), and history of a diagnosed psychological condition or medical condition relevant for COVID-19 severity (Sherman et al., 2020). We also measured psychological symptoms that were credited to be relevant in the context of COVID-19, vaccination, and mistrust/conspiracy beliefs, i.e., anxiety and depression (Kar et al., 2021), death and disease anxiety (Simione and Gnagnarella, 2020), somatization (Shigemura et al., 2020), anger (Trnka and Lorencova, 2020), paranoid ideation (Lopes et al., 2020), and psychotic symptoms (Hajdúk et al., 2020; D′Agostino et al., 2021). Lastly, we designed a new inventory that probed the presence of belief in conspiracy theories related to COVID-19, mistrust in science and in policy makers, and mistrust in scientific information on the pandemic.

Following the literature review presented, we hypothesized that psychological variables would influence the mistrust/conspiracy beliefs factors, and these, in turn, would reduce the propensity to get vaccinated. To test this hypothesized relationship scheme, we developed a mediation model in which the psychological factors of stress, general distress, anxiety, death anxiety, paranoia, psychoticism, somatization, and anger were the predictors, mistrust/conspiracy belief/misinformation factors were the mediators, and vaccine propensity was the outcome. In particular, we hypothesized that anxiety would increase vaccine propensity by increasing trust in medical science (Mannan and Farhana, 2020), whereas stress would lead to increased adherence to conspiracy beliefs and in turn to increased vaccine hesitancy (van Prooijen and Douglas, 2018). Psychopathological symptoms such as paranoid ideation, psychoticism, and hostility/anger would increase mistrust/conspiracy belief/misinformation and thus decrease vaccine propensity (Imhoff and Lamberty, 2018; Georgiou et al., 2019; Murphy et al., 2021). The presence of psychosomatic symptoms would increase health worries related to COVID-19 infection (Grönros et al., 2020), thus increasing the vaccine propensity. Lastly, death anxiety would increase the propensity to get vaccinated (Patelarou et al., 2021), whereas, on the contrary, a mistrust and suspicious position (van Prooijen, 2020) could decrease the vaccine propensity.

## Materials and Methods

### Participants

We enrolled 374 Italian adults for this study. We removed 24 participants as multivariate outliers (see section Data Analysis for details), and we obtained a final sample of 350 participants for the analysis. Descriptive statistics of the sample are reported in Table 1. This sample included 292 females (81%) and 58 males (19%), with a mean age of 40.77 years (SD = 10.74) and a mean education level of 15.07 years (SD = 4.10). Of our participants, 52 (15%) reported working as medical doctors or in the healthcare system. Of our participants, 52 (15%) reported working as medical doctors or in the healthcare system, and 246 (70%) reported they were in a relationship. With regard to religious belief, 105 (30%) reported to be atheist or agnostic, 164 (47%) to be non-practicing Catholics, and 81 (23%) to be practicing Catholics. With regard to psychological and medical conditions, 31 (9%) reported having one or more psychiatric disorders such as depression or anxiety, while 67 (19%) reported at least one medical condition associated with an increased risk in the event of COVID-19 infection (mean = 1.40). While our sample was unbalanced for sex (81% of females), we conducted a series of Holm's corrected two-sample *t*-tests in order to assess differences between females and males in the other measured variables. The analysis revealed that, with respect to males, females reported on average lower education level, lower presence of smart working, and higher religious beliefs. No difference emerged for the other variables.

**Table 1:** Descriptive statistics for the demographic and psychological variables (*N* = 350).

**Demographic variables**	**Yes (1)**	**No (0)**	
In smart working	30%	70%	
Healthcare worker	15%	85%	
In a relationship	70%	30%	
Psychological condition	9%	91%	
	**M**	**SD**	
Age	40.77	10.74	
Education (in years)	15.07	4.1	
Number of children	1.06	0.95	
Religious beliefs (0 = atheist, 1 = non-practicing, 2 = practicing)	0.93	0.73	
Medical conditions relevant for COVID-19	0.27	0.62	
**Psychological variables**	**M**	**SD**	**Cronbach's** **α**
PSS	19.59	6.95	0.81
STAI	14.3	4.33	0.87
ECQ	8.72	5.66	0.90
GHQ	18.14	5.79	0.83
SCL-90 somatization	14.43	10.98	0.91
SCL-90 anger	4.71	4.41	0.85
SCL-90 psychoticism	5.62	6.31	0.84
SCL-90 paranoid ideation	5.1	4.9	0.81

*M, mean; SD, standard deviation. Healthcare worker is coded as 0 = no, 1 = yes; In a relationship is coded as 0 = no, 1 = yes; Religious belief is coded as 0 = atheist/agnostic, 1 = non-practicing, 2 = practicing; Psych. condition is coded as 0 = no, 1 = yes; Med. condition is coded from 0 to 8*.

### Procedure

The entire procedure was administered through online forms. In the first form, participants read the informed consent and gave their agreement to participate. In the second form, we collected demographic information. Then, a series of questionnaires was presented in successive online forms, in the same order as reported in Materials and Methods section. All data were collected in a completely anonymous format. Ethical approval for this study was granted by the Research Ethics and Integrity Committee of CNR, and all procedures performed were in accordance with the ethical standards of the 1964 Helsinki Declaration.

### Materials

We administered a battery of questionnaires to the participants, including measures for stress, anxiety, death anxiety, psychological distress, psychoticism, paranoid ideation, anger, and somatization. Prior to assessing psychological data, we collected demographic information, including sex, age, education level, if in a relationship, if working as a healthcare worker, religious belief, presence of psychological or psychiatric conditions, and presence of medical conditions associated with increased risk in the event of COVID-19 infection. The latter measure was computed as the raw sum (from 0 to 8) of eight possible conditions measured by means of a checklist, including cardiovascular diseases, diabetes mellitus, hypertension, chronic pneumopathies, neoplasms, immunodeficiencies, hematological pathologies, and neuromuscular diseases.

With regard to psychological conditions (see Table 1 for descriptive statistics), we administered the 10-item Perceived Stress Scale (PSS; Cohen et al., 2006), assessing a total score measuring how respondents perceive their lives as unpredictable and overloaded. In our sample, PSS showed a good internal reliability, Cronbach's α = 0.81. In measuring anxiety, we used a short version of the State-Trait Anxiety Scale (STAI; Marteau and Bekker, 1992), using only six items. This scale showed an excellent internal reliability in our sample, Cronbach's α = 0.87. Then, we measured death anxiety as a fear of death, illness, and in general of the unpredictability of life. We administered the 5-item subscale of the death anxiety scale of the Existential Concerns Questionnaire (ECQ; Van Bruggen et al., 2017), which showed an excellent internal reliability, Cronbach's α = 0.90. We also measured general distress and depression symptoms by means of the 12-item version of the General Health Questionnaire (GHQ; Giorgi et al., 2014). We computed a total score from the 12 items, showing a good internal reliability in our sample, Cronbach's α = 0.83.

In measuring psychological symptoms, we administered four subscales of the Symptom Checklist (SCL-90; Prunas et al., 2012). In particular, we used the 10-item subscale for psychoticism (as the presence of social withdrawal, isolation, and schizoidia), the 6-item subscale for paranoid ideation (as the presence of suspect and ideas of reference), the 6-item subscale for hostility/anger (as the presence of anger, irritability, and resentment), and the 12-item subscale for somatization (as the presence of perception of bodily dysfunctions and somatic concerns). All these measures showed good to excellent internal reliability in our sample, with Cronbach's α ranging from 0.81 to 0.91.

Lastly, we administered the items that we designed about Beliefs on COVID-19 (BOC-19). A first version of the inventory was created by the authors LS and CG. This first version had 16 items, including four items for each of the following dimensions: beliefs on conspiracy theories about COVID-19, reaction to communication from experts and virologists, mistrust in medicine, and mistrust in policymakers. Then, the inventory was revised by the co-authors and a group of six external experts (clinical psychologists and psychiatrists), who proposed removing three items as replication of other items or for unclear contents, leading to a final set of 12 items. Thus, the final version of the inventory included four items (numbers 1, 2, 3, and 4) investigating beliefs about conspiracy theories regarding COVID-19, three items (numbers 6, 7, and 11) investigating problems and misunderstandings in communication from experts and virologists, three items (numbers 5, 8, and 9) investigating mistrust in scientific research and medical science, and two items (numbers 10 and 12) investigating mistrust in policymakers and health systems. Each item consisted of a statement (e.g., “The new coronavirus responsible for COVID-19 was created artificially”) that participants had to rate on a 5-point Likert scale ranging from 1 (completely disagree) to 5 (completely agree). Together with these items, we administered a vaccine propensity item (“If a vaccine were available for COVID-19, I would get vaccinated”) measured on the same 5-point Likert scale.

### Data Analysis

First, we computed the scores from the raw scale values. For each score, we computed descriptive statistics such as mean, standard deviation, and reliability as Cronbach's α. Before running the main analysis, we checked for multivariate outliers on the measured psychological scales by means of Cook's distance (Fox, 2016) and in this way excluded 24 participants as outliers. We also checked for the presence of a common method bias through Harman's one-factor test (Podsakoff et al., 2003) and the correlation matrix procedure (Bagozzi et al., 1991).

We then conducted the EFA on the BOC-19 inventory in order to assess the structure of the scale in our sample. For this analysis, we used the method of ordinary least squares (OLS) to find the minimum residual solution, and we applied an oblique rotation (oblimin) that assumes factors are correlated. We estimated the number of factors to be extracted with both scree analysis and BIC values testing solutions including 1–5 factors. We evaluated the suggested solutions by means of their factor structure together with their goodness of fit (Preacher et al., 2013). In particular, the model-fitting indexes include χ2 statistics, Tucker–Lewis index (TLI), root mean square of the residuals (RMSA), and root mean square error of approximation (RMSEA) with related 90% confidence intervals. Model fit was considered as adequate with the following values: non-significant χ2, RMSEA of 0.06 or less, SRMR of 0.08 or less, CFI and TLI above 0.95 (Hu and Bentler, 1999).

We then moved on to the mediation model and to the related diagnostic regression analyses. Psychological variables of stress, anxiety, death anxiety, general distress, psychoticism, paranoia, hostility/anger, and somatization were predictors, while the outcome was the propensity to get vaccinated. The BOC-19 factors were mediators; thus, they were considered as dependent variables (predicted by psychological factors) or as predictors (of the vaccine propensity) in the diagnostic regression analyses. We considered our demographic variables as possible covariates in our regression models.

We tested the hypothesized mediational pathways through structural equation modeling (Kline, 2011) as it is considered a better method for assessing mediation with respect to regression methods (Iacobucci et al., 2007). We conducted model analysis by means of maximum-likelihood estimation, and we reported both unstandardized (with its relative confidence intervals) and standardized coefficients. Parameters of both regressions and SEM were estimated by means of bootstrapping over 1,000 samples, because it is considered the best method to make a model fitting robust to non-normal data (Mooney and Duval, 1993; Lai, 2018). We also tested the model using the Huber–White robust standard errors estimator in order to exclude bias due to heteroscedasticity. We used bias-corrected bootstrapped confidence intervals to test the indirect effects of psychological variables on vaccine propensity through BOC-19 factors. Confidence intervals were reported with each estimated coefficient and its related test of significance.

As we obtained a consistent number of candidate predictors, we proceed with variable selection prior to fitting the mediation model. Then, in the final model, we only included the variables that correlated with the predictors, the dependent variables, or both (VanderWeele, 2019). In order to assess if the exclusion of some variables might affect the model statistics, we compared a complete model (including all the considered variables) with a set of candidate nested models in which one or more variables or paths were removed. Model comparison was conducted by means of Akaike Information Criterion (AIC) and Bayesian Information Criterion (BIC) values, and the final model selection relied on their weight (Burnham and Anderson, 2002), i.e., the relative difference in criterion between the better model and the worst one.

All our analyses were conducted with R statistical software (R Core Team, 2014). In particular, we evaluated our regression and mediation models by means of the Lavaan package (Rosseel, 2012).

## Results

We checked for the presence of a common method bias with the Harman's one-factor test (Podsakoff et al., 2003). Thus, we computed the variance explained by a single-factor exploratory model including all the items administered. The proportion of variance explained by this single-factor model was only 22.17%, suggesting the absence of a bias (test critical threshold = 50%). We also conducted the correlation matrix test that confirmed the absence of response bias, as all the correlation coefficients between our variables were smaller than the critical threshold value of 0.90.

### Factor Structure of the Belief on COVID-19 Scale

First, we conducted the EFA on the Beliefs of COVID-19 (BOC-19) scale. The parallel analysis suggested the presence of three factors and the scree plot showed that only two factors had an eigenvalue higher than 1. Thus, we tested both a 2-factor and a 3-factor solution via a minimum residual algorithm and an oblimin rotation. Table 2 reports the factor loadings for the two solutions, with their relative fit indices. As shown, both models explained about half of the total variance, respectively, 0.46 and 0.51 for the 2-factor and the 3-factor model. However, only the 3-factor model showed satisfactory fit indices, χ^2^(43) = 67.08, *p* < 0.05, TLI = 0.97, RMSA = 0.03, RMSEA = 0.05, CI_RMSE_ = [0.03, 0.07], while the 2-factor model did not, χ^2^(33) = 384.90, *p* < 0.05, TLI = 0.76, RMSA = 0.06, RMSEA = 0.15, CI_RMSE_ = [0.13, 0.16], whit RMSEA > 0.06 and TLI < 0.95. The two solutions differed in the factor that included two items about information confusion (i.e., items 6 and 7), whereas the other items loaded in the very same factors, and with the item 12 did not load significantly in any factor for both models. Thus, we decided to use the 3-factor model for the successive analysis.

**Table 2:** Exploratory factor analysis results: oblimin-rotated factor loadings and explained variance for the two alternative models.

	**2-factor model**		**3-factor model**		
**Item**	**F1**	**F2**	**F1**	**F2**	**F3**
1. The new coronavirus responsible for COVID-19 was created artificially.		0.84	0.84		
2. The new coronavirus responsible for COVID-19 was spread voluntarily by some entity or person.		0.94	0.94		
3. The spread of COVID-19 is due to the use of innovative technologies without proper verification of their effects on health.		0.79	0.79		
4. There are effective treatments that the population does not know.		0.49	0.53		
5. I think that research and medical science are not capable of giving us adequate measures to deal with COVID-19.	0.49				0.32
6. Information on COVID-19 provided by virologists and official sources changes constantly and is unclear.	0.80			1.01	
7. Virologists and other experts have very different opinions on COVID-19; thus, it is difficult to understand which one is the best strategy to adopt.	0.75			0.84	
8. I do not trust the international scientific community and in medical research.	0.61				0.54
9. Healthcare system is dealing too much with the COVID-19 emergency to the detriment of the needs of care of other patients.	0.62				0.78
10. Important public health decisions should be made with greater collaboration between the experts and the general population.	0.48				0.39
11. Doctors and healthcare professionals should pay more attention to the emotional impact of their communications.	0.64				0.56
12. Experts and policy makers are forced to impose their decisions on the population as they are unable to regulate themselves.					
Proportion of variance explained	0.25	0.22	0.22	0.16	0.13
Cumulative variance explained	0.25	0.46	0.22	0.38	0.51

*Loadings below.30 are not shown. F1, F2, and F3 refer to the different factors in each model*.

The first factor included items 1, 2, 3, and 4, which investigated the beliefs on conspiracy theories of COVID-19. The second factor included items 6 and 7, which investigated the confusion about the information given about COVID-19 by virologists and medical doctors. The third and last factor included items 5, 8, 9, 10, and 11, which investigated the two topics of the mistrust in science reaction to COVID-19 and the inability of the healthcare system to manage the situation. Therefore, we called the first factor “belief in conspiracy theories” (BCT), the second factor “mistrust in medical information” (MMI), and the third factor “mistrust in medicine and science” (MMS). We then computed the values for the three scales for all the participants. As expected, the three scales were highly and positively correlated, with BCT correlated with MMI, *r* = 0.40, and MMS, *r* = 0.45, and MMI correlated with MMS, *r* = 0.52.

### Correlation Analysis With Demographic and Psychological Variables

We then computed the Pearson bivariate correlations between the three BOC-19 scales and the demographic variables. The results of the analysis in terms of correlation coefficients are reported in Table 3. As reported, female sex was correlated with higher scores on both BCT and MMS scales, age was weakly correlated with a higher score on MMS scale, education was highly and negatively correlated with all the BOC-19 scales, religious belief was positively correlated with BCT and MMS scores, and reporting a psychic condition was positively correlated with BCT score. Working in the healthcare system, being in a relationship, and having one or more medical pathologies increasing risk in the event of COVID-19 infection did not correlate significantly with any of the scores. Thus, we considered only the variables significantly related to at least one of the BOC-19 scales as covariates in the successive regression models.

**Table 3:** Correlation coefficients of BOC-19 factors with sociodemographic and psychological variables.

	**BOC-19 factors**		
**Variable**	**BCT**	**MMI**	**MMS**
Sex	0.20^**^	0.10	0.14^*^
Age	−0.02	0.05	0.14^**^
Education	−0.48^**^	−0.27^**^	−0.24^**^
Healthcare worker	−0.10	−0.08	0.01
In a relationship	0.02	0.01	−0.04
Religious belief	0.21^**^	0.09	0.15^**^
Psyc. condition	0.16^**^	0.10	0.08
Med. condition	−0.01	−0.04	0.03
PSS	0.20^**^	0.23^**^	0.16^**^
STAI	0.14^**^	0.22^**^	0.11^*^
ECQ Death anxiety	0.26^**^	0.27^**^	0.22^**^
GHQ	0.19^**^	0.26^**^	0.27^**^
SCL90 Somatization	0.28^**^	0.24^**^	0.20^**^
SCL90 Anger/hostility	0.10	0.20^**^	0.09
SCL90 Psychoticism	0.29^**^	0.24^**^	0.25^**^
SCL90 Paranoid ideation	0.25^**^	0.25^**^	0.24^**^

*Sex is coded as 0* = *male, 1* = *female; Healthcare worker is coded as 0* = *no, 1* = *yes; In a relationship is coded as 0* = *no, 1* = *yes; Religious belief is coded as 0* = *atheist/agnostic, 1* = *non–practicing, 2* = *practicing; Psych. condition is coded as 0* = *no, 1* = *yes; Med. condition is coded from 0 to 8. BCT, Belief in conspiracy theories; MMI, Mistrust in medical information; MMS, Mistrust in medicine and science. Significant levels are reported as follows*

* *p < 0.05*,

** *p < 0.01*.

We also computed the correlation coefficients between the three BOC-19 scales and the psychological variables measured. The results of this analysis are reported in Table 3. As shown, all the reported psychological variables had at least one positive correlation with a BOC-19 scale. In particular, death anxiety, somatization, psychoticism, and paranoia showed high correlations with all the BOC-19 scales, whereas anxiety (STAI) and anger/hostility showed low or nonsignificant correlations with BCT and MMS scales.

### Vaccine Propensity: Mediation Effect of BOC-19 Scales

**Figure 1:**
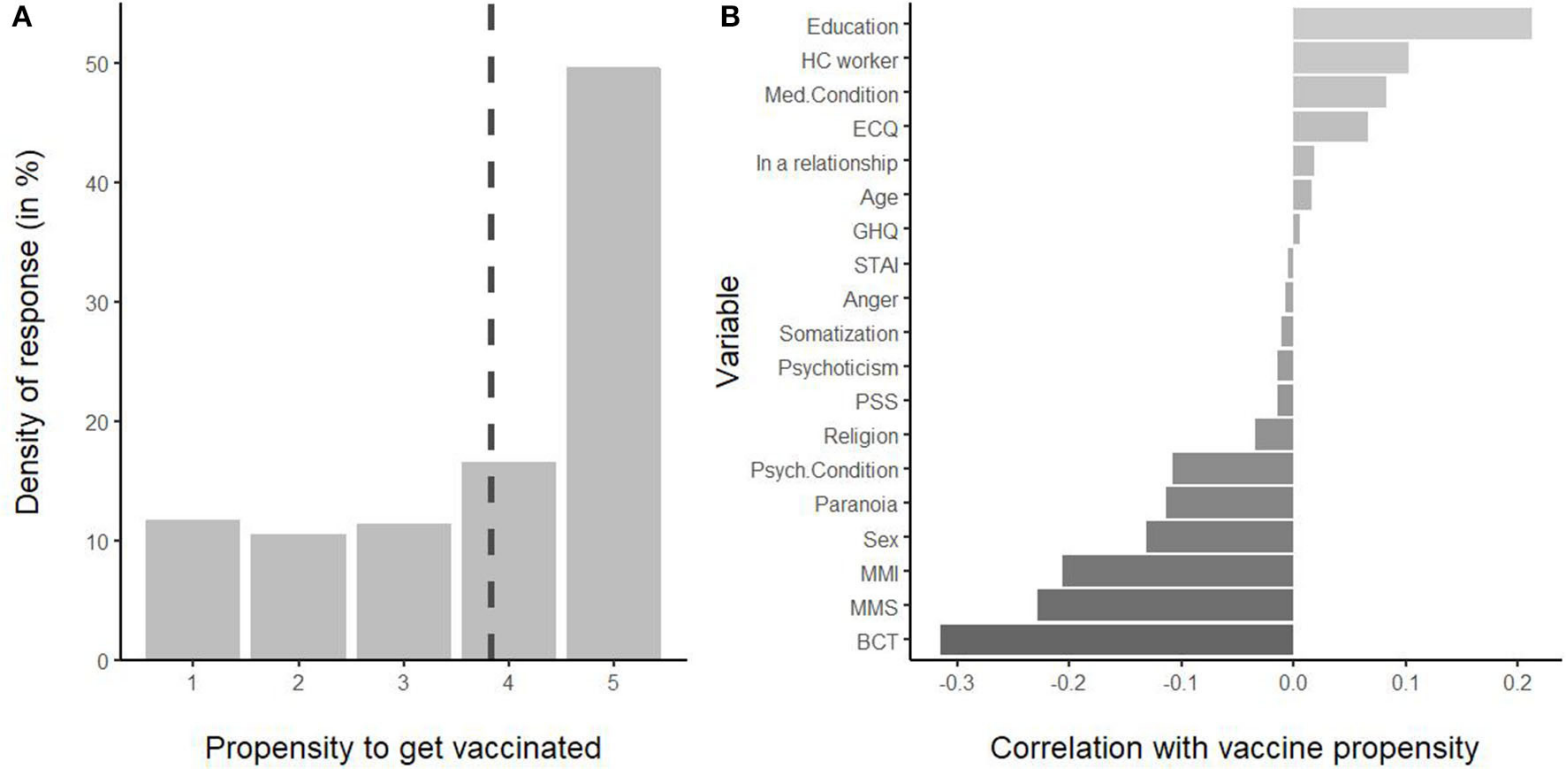
**(A)** Distribution of responses for the vaccine propensity. The dashed line indicates the mean. **(B)** Correlation coefficients of vaccine propensity with demographic and psychological variables, ordered by coefficient. HC worker, healthcare worker; BCT, belief in conspiracy theories; MMI, mistrust in medical information; MMS, mistrust in medicine and science.

After the factor and correlation analysis, we assessed the pattern of the relationship between psychological variables, beliefs about COVID-19, and propensity to get vaccinated against SARS-COV-2. First, we conducted a descriptive analysis of vaccine propensity (VP) and its correlation with demographic and psychological variables. As reported in the panel A of Figure 1, about half of the participants declared they fully agreed with getting vaccinated against COVID-19. The mean value was 3.82, significantly higher than the midpoint of the scale, *t*_(349)_ = 10.68, *p* < 0.01. Vaccine propensity positively correlated with education level, working in the healthcare system, and the presence of a medical condition, whereas it negatively correlated with the presence of a psychological condition, paranoid ideation, female sex, MMI, MMS, and BCT. The other variables were not significantly related to vaccine propensity.

**Figure 2:**
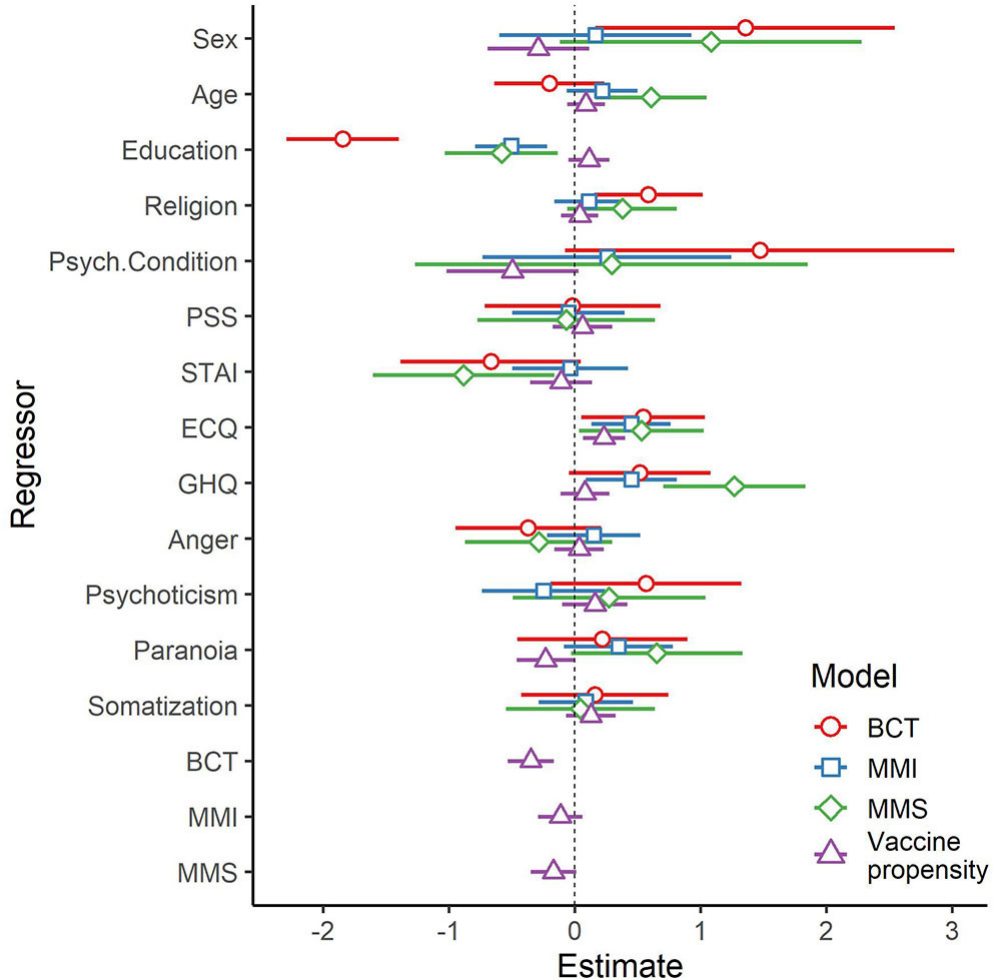
Regression coefficients with 95% confidence intervals for the four models tested. BCT, belief in conspiracy theories; MMI, mistrust in medical information; MMS, mistrust in medicine and science.

Before testing the mediation model, we conducted diagnostic regression analyses testing the relationship between demographic and psychological variables as predictors, the three BOC-19 scales as mediators, and vaccine propensity, as the outcome. We included in the regression models the covariates that showed at least one significant correlation with any of the BOC-19 scales, i.e., sex, age, education, religion, and presence of a psychological condition. We also included all the psychological scales measured, i.e., PSS, STAI, ECQ death anxiety, GHQ, somatization, anger/hostility, psychoticism, and paranoid ideation (reported as paranoia). In the model with vaccine propensity as a dependent variable, we also included the three BOC-19 factors as regressors, i.e., BCT, MMI, and MMS. We aimed at identifying the significant relationships between the variables in order to select the ones to be included in the final mediation model. The results of this analysis are reported in Table 4, which included for each regressor the unstandardized coefficient (b) with its bootstrapped 95% confidence intervals and the semi-partial correlation (*sr*) as interpretable measures of effect size. We also reported the same coefficients in Figure 2, which compares all the four models for each predictor.

**Table 4:** Regression results for the three BOC-19 scales and vaccine propensity.

	**BCT**				**MMI**				**MMS**				**Vaccine propensity**			
	***b***	**95% CI**		***sr***	***b***	**95% CI**		***sr***	***b***	**95% CI**		***sr***	***b***	**95% CI**		***sr***
**Regressor**		**LL**	**UL**			**LL**	**UL**			**LL**	**UL**			**LL**	**UL**	
(Intercept)	14.02^**^	11.00	16.79		5.13^**^	2.83	7.85		8.22^**^	5.11	11.28		2.59^**^	1.60	3.65	
**Sex**	1.36^*^	0.47	2.30	0.10	0.17	−0.49	0.77	0.02	1.08	−0.35	2.44	0.09	−0.44^*^	−0.70	−0.03	−0.11
**Age**	−0.02	−0.05	0.02	−0.04	0.02	−0.01	0.05	0.08	0.06^**^	0.02	0.09	0.13	0.01	−0.01	0.02	0.05
**Education**	−0.45^**^	−0.53	−0.36	−0.36	−0.12^**^	−0.20	−0.06	−0.17	−0.14^*^	−0.24	−0.02	−0.12	0.07^**^	0.04	0.12	0.19
**Religion**	0.81^**^	0.24	1.39	0.12	0.16	−0.23	0.57	0.04	0.52	−0.03	1.04	0.08	−0.03	−0.22	0.18	−0.01
**Psych. Cond**.	1.47	−0.29	3.67	0.08	0.26	−0.46	1.09	0.03	0.29	−1.18	2.31	0.02	−0.62^*^	−1.04	−0.06	−0.12
PSS	0.01	−0.08	0.10	0.01	−0.01	−0.07	0.04	−0.01	−0.01	−0.09	0.09	−0.01	0.01	−0.01	0.06	0.03
**STAI**	−0.15	−0.32	−0.01	−0.08	−0.01	−0.10	0.11	−0.01	−0.20^*^	−0.35	−0.02	−0.12	−0.01	−0.06	0.06	−0.01
**ECQ**	0.10^*^	0.01	0.21	0.10	0.08^**^	0.03	0.14	0.14	0.09^*^	0.03	0.17	0.10	0.04^*^	0.01	0.07	0.09
**GHQ**	0.09	0.00	0.19	0.08	0.08^*^	0.01	0.15	0.12	0.22^**^	0.12	0.31	0.21	−0.00	−0.03	0.03	−0.01
Anger	−0.08	−0.18	0.05	−0.06	0.03	−0.03	0.12	0.04	−0.06	−0.19	0.05	−0.05	0.02	−0.03	0.06	0.03
Psychoticism	0.09	−0.03	0.24	0.07	−0.04	−0.11	0.03	−0.05	0.04	−0.08	0.13	0.03	0.02	−0.01	0.05	0.05
**Paranoia**	0.05	−0.05	0.18	0.03	0.07	−0.02	0.15	0.08	0.13^*^	0.02	0.27	0.09	−0.06^*^	−0.10	−0.02	−0.12
Somatization	0.01	−0.03	0.07	0.02	0.01	−0.02	0.05	0.02	0.01	−0.06	0.07	0.01	0.01	−0.00	0.03	0.06
**BCT**	–				–				–				−0.07^**^	−0.11	−0.04	−0.19
MMI	–				–				–				−0.04	−0.10	0.03	−0.06
**MMS**	–				–				–				−0.04^*^	−0.08	0.00	−0.09
Model fit	*R*^2^ = 0.33^**^				*R*^2^ = 0.17^**^				*R*^2^ = 0.21^**^				*R*^2^ = 0.18^**^			

*Boldface predictors indicate variables with at least a significant effect. A significant b-weight indicates the semi-partial correlations are also significant. b represents unstandardized regression weight, with 95% CIs. LL and UL indicate the lower and upper limits of a confidence interval, respectively. sr represents the semi-partial correlation. BCT, Belief in conspiracy theories; MMI, Mistrust in medical information; MMS, Mistrust in medicine and science. Significant levels are reported as follows*

a * *p < 0.05,*

** *p < 0.01*.

With regard to the covariates, education showed an effect on all the BOC-19 scales (positive) as well as vaccine propensity (negative). In addition, sex increased BCT and reduced vaccine propensity, age increased MMS, religion increased BCT, and the presence of a psychological condition decreased the propensity to get vaccinated. Thus, all the covariates showed at least one significant effect on the dependent variables tested. With regard to the psychological factors, the ECQ death anxiety was a significant positive predictor for all the models tested. The GHQ significantly increased MMI and MMS, while the STAI decreased MMS. Paranoia increased MMS and decreased the propensity to get vaccinated. The other factors, i.e., PSS, somatization, anger, and psychoticism, did not significantly relate to any dependent variables.

Our last analysis was the multiple mediation model. We tested all the direct and indirect paths considered by means of a structured equation model with parameters estimated on 1,000 bootstrapped samples. Before conducting the final analysis, we compared nested models including a different set of variables (and relative paths). First, we fitted a “complete model” including all the measured psychological variables and the three BOC-19 factors as mediators, with vaccine propensity as an outcome variable. Then, we fitted a “minimal model” including only the variables that should be retained based on our diagnostic regression analyses, i.e., ECQ, GHQ, STAI, and paranoia as psychological factors, and BCT and MMS as mediators (MMI did not affect vaccine propension; see Table 4). As we were particularly interested in the measures we developed, we also fitted a third model (“full BOC-19 model”) including the same psychological factors of the minimal model but all the three BOC-19 factors as mediators. All the three models were fully saturated, so model fit could not be assessed. We then compared the models' AIC and BIC values, considering their likelihood while penalizing unnecessarily complex models. The models AIC were 6679.52, 6662.93, and 5099.47, respectively, for the complete, the full BOC-19, and the minimal model; the models BIC were 6918.71, 6840.39, and 5226.78, respectively, for the complete, the full BOC-19, and the minimal model. As shown, the minimal model outperformed both the complete and the full BOC-19 models, with Δ_AIC_ > 1,563 and Δ_BIC_ > 1,613, and thus AIC weights < 0.01 for both non-minimal models. This analysis suggested that removing the psychological variables of PSS, somatization, anger, and psychoticism (complete model) and the BOC-19 factor of MMI (full BOC-19 model) led to a model that was both simpler and closer to the true model.

**Figure 3:**
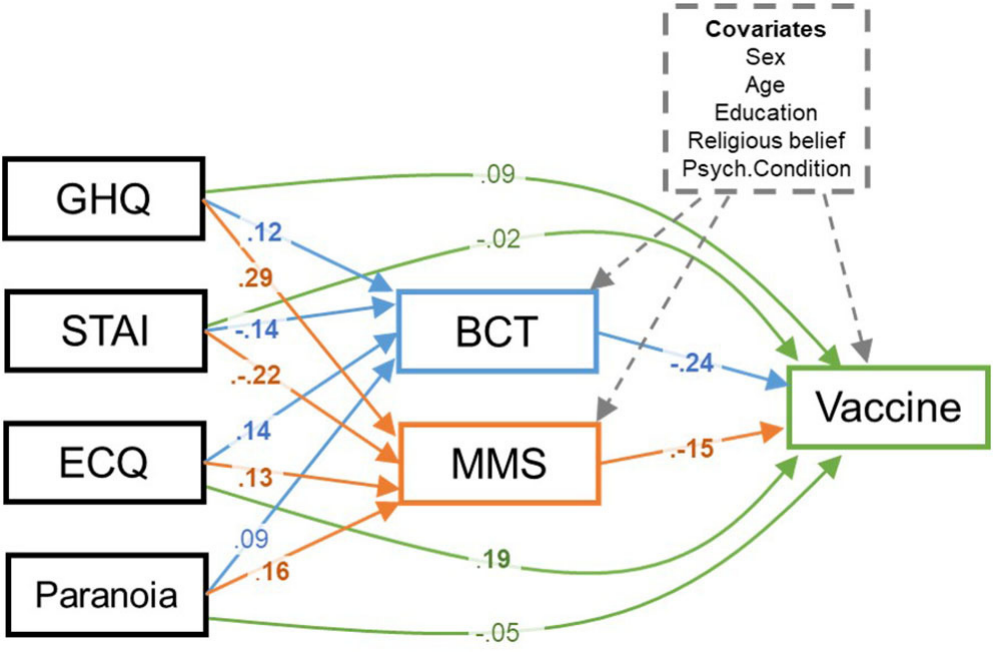
The structured equation model tested. Standardized coefficients are reported only for direct paths. Coefficients for significant paths were reported in the boldface. Indirect effects are reported in text. BCT, belief in conspiracy theories; MMS, mistrust in medicine and science; Vaccine, propensity to get vaccinated.

Following all these preliminary analyses, we tested a final model (see Figure 3) that included four covariates, i.e., sex, age, education, and religious belief; four psychological predictors, i.e., death anxiety (ECQ), GHQ, STAI, and paranoia; two BOC-19 mediators, i.e., BCT and MMS factors; and vaccine propensity as a dependent variable. The analysis revealed that BCT was significantly increased by ECQ (*b* = 0.12, *CI* = [0.04, 0.20], β = 0.14), and GHQ (*b* = 0.10, *CI* = [0.01, 0.18], β = 0.12), while it was significantly decreased by STAI (*b* = −0.16, *CI* = [−0.28, −0.02], β = −0.14). MMS was significantly increased by ECQ (*b* = 0.10, *CI* = [0.01, 0.19], β = 0.13), GHQ (*b* = 0.22, *CI* = [0.13, 0.31], β = 0.29), and paranoia (*b* = 0.14, *CI* = [0.03, 0.23], β = 0.16), while it was significantly decreased by STAI (*b* = −0.22, *CI* = [−0.36, −0.08], β = −0.22). Negative effect on the propensity to get vaccinated was significant for the path from both BCT (*b* = −0.07 6, *CI* = [−0.12, −0.03], β = −0.24) and MMS (*b* = −0.05, *CI* = [−0.09, −0.01], β = −0.15), whereas it was significantly and directly increased by ECQ (*b* = 0.05, *CI* = [0.02, 0.08], β = 0.19).

The test of indirect effect through bias-corrected bootstrapped CI showed that STAI had a mediated positive effect on vaccine propensity through a reduction in BCT (*b* = 0.01, *CI* = [0.01, 0.03], β = 0.03) and that ECQ had a mediated negative path through an increase in BCT (*b* = −0.01, *CI* = [−0.02, −0.01], β = −0.04). Instead, both GHQ (*b* = −0.01, *CI* = [−0.02, −0.01], β = −0.04) and paranoia (*b* = −0.01, *CI* = [−0.02, −0.01], β = −0.02) decreased the propensity to get vaccinated by increasing the MMS factor.

The covariates showed a similar pattern of results as revealed by the previous regression analyses for their effect on mediators. In fact, BCT was increased by female sex (*b* = 1.23, *CI* = [0.31, 2.21], β = 0.10) and religious belief (*b* = 0.87, *CI* = [0.31, 1.45], β = 0.13), while it was reduced by higher education level (*b* = −0.46, *CI* = [−0.55, −0.35], β = −0.40); MMS increased with age (*b* = 0.06, *CI* = [0.02, 0.10], β = 0.15) and decreased with education level (*b* = −0.15, *CI* = [−0.25, −0.03], β = −0.14). However, none of the covariates significantly related to vaccine propensity in this model.

We also fitted this model through a robust model estimator, i.e., the maximum-likelihood estimation with robust (Huber–White) standard errors. The result from this control analysis showed no difference with our main analysis using the bootstrapped samples; thus, we could conclude that no bias from non-normal or outlier data affected the results.

## Discussion

In this paper, we designed and tested an inventory for measuring beliefs about COVID-19 and their relationships with the propensity to get vaccinated. We collected data from a convenience sample of the general population, together with data on psychological symptoms and distress. We showed that three main areas emerged from the factorial analysis of the proposed inventory, i.e., believing in conspiracy theories about COVID-19, mistrust in medicine and scientific research, and mistrust in medical information about COVID-19 from experts and virologists. While these factors were all positively correlated with each other, they should be considered as distinct dimensions. The three scores were higher in female participants and in the presence of religious beliefs, whereas they decreased with an increased level of education. As female sex was positively correlated with religious belief and negatively correlated with education level, these effects could be explained by a common factor not included in the present model. Moreover, they were all positively related to the presence of psychological symptoms, in particular to death anxiety and paranoid ideation. We then conducted a mediation model, in which we included all the factors that survived the diagnostic regression models, i.e., psychological distress, anxiety, death anxiety, and paranoia, with covariance factors of age, sex, education level, religious belief, and presence of a psychological condition. We tested this model by means of structural equation modeling, and we found that death anxiety reduced the propensity to get vaccinated through a mediated path in believing in conspiracy theories, whereas paranoia was linked to a reduction in vaccination adherence with the mediation effect of mistrust in medical science. On the contrary, anxiety increased the propensity to get vaccinated through a decrease in both belief in conspiracy theories and mistrust in science. Lastly, death anxiety also had a direct positive effect on the propensity to get vaccinated. Thus, our study showed how psychological dimensions differentially relate to the belief in conspiracy theories, to mistrust in science, and to the propensity to get vaccinated. In particular, our data suggested a predominant effect of death anxiety on believing in conspiracy theories, while paranoia was the principal determinant of mistrust.

### Mistrust and Conspiracy Beliefs Are Correlated but Distinct Factors

As shown, the three factors were strongly correlated with each other, suggesting that people who believe in conspiracy theories also tend not to trust science or medicine. In fact, a recent study on COVID-19 conspiracy theories showed that they are linked to denialism toward official sources of information, such as medical doctors or experts (Uscinski et al., 2020). This could be also explained by the tendency for conspiracy theories believers to self-feed, so that the more people are involved with those theories, the more they stick to them (van Prooijen, 2020). This reveals a typical echo chambers dynamics, where ignoring information from experts or official channels is a strategy to protect or maintain a core of beliefs that are functional for the individual (Uscinski et al., 2020). Skepticism toward science and policymakers could also lead to conspiracy beliefs acceptance, in particular when a group credited as “responsible” can clearly be identified (van Prooijen, 2020), e.g., the Chinese government or Big Pharma, or if one is involved in a conspiracy online group, in which the sense of isolation and trust in conspiracy theories tends to increase during time (Del Vicario et al., 2016).

Thus, each one of these attitudes or beliefs about COVID-19 is correlated with psychological ill-being and is interconnected to the other beliefs in a complex and dynamic way. In fact, BCT seems more related to religious belief and existential anxiety, while mistrust (MMI and MMS) seems more related to distress and anxiety, with a special role for paranoia. In light of this result, BCT may be more linked to fear of death and disease, i.e., to deep existential concern (as suggested also in van Prooijen, 2020) or to a sense-making motivation (Park, 2010), whereas MMS seems to be more linked to psychological ill-being and negative emotional state. Having a paranoid or suspicious stance was a predictor of MMS, but not of BCT.

Based on our results, we can propose a new hypothesis in the interrelation between these constructs. First, an individual with a paranoid attitude, in the presence of an important stressful event, starts to lose trust in agencies and organizations that are considered to be incapable or incompetent, e.g., a person gets fired during the pandemic spread because the government proposed containment measures. Then, the same person finds information against the distrusted agencies and eventually gets involved in an online group of skeptics. In the presence of existential concerns, this person starts to search for a sense of what is happening, that is, experienced as uncertain or out of control. Moreover, the judgment on the same agencies and organizations would change from incompetent to hostile. This feeling grows as the person is even more isolated in a conspiracy echo chamber and overwhelmed by his or her negative emotional state. In this way, mistrust and misinformation interact with stress, paranoid ideation, and existential anxiety in determining the formation and the defense of conspiracy theories.

### Direct and Indirect Effects of Psychological Factors on Vaccine Propensity

In our sample, we found a moderate propensity to get vaccinated for COVID-19 that is in line with a recent global survey (Mannan and Farhana, 2020). In the regression models, vaccine propensity decreased for females with respect to males and increased with education levels, according to previous studies on this topic (Lazarus et al., 2020; Malik et al., 2020). Moreover, existential anxiety and general anxiety increased vaccine propensity, while it was reduced in the presence of paranoid ideation. Again, these results are in line with previous literature showing that anxiety and fear may be associated with higher trust in COVID-19 vaccination (Mannan and Farhana, 2020; Patelarou et al., 2021).

Interestingly, we found no direct effect of anger, somatization, and perceived stress on vaccine acceptance in the regression model. About anger, we should consider that hostility tends to increase over time as distress increased and lockdown measures persisted (Duan et al., 2020). As we conducted this survey in the very first period of pandemic spread, it is possible that at that time anger was not a prominent factor for influencing vaccine intention. The absence of effect for somatization was also unexpected, but it could be explained as people with high psychosomatic symptoms could be equally scared by both COVID-19 (Grönros et al., 2020), thus propending for vaccination, but also by the vaccine's side effects, thus refusing to take the vaccine (as suggested also in Mannan and Farhana, 2020). Lastly, stress was also not related to COVID-19 vaccine intention. A previous study suggested a link between these two factors, but it tested a sample of nurses, a population at high risk of infection (Kwok et al., 2020). Thus, while stress could have a role, vaccine propensity seems more related to fear of disease and death, or to health worries (as also shown in Pastorino et al., 2021).

Out of the three factors of BOC-19, only BCT and MMS were related to vaccine propensity, whereas MMI was not. While misinformation was usually related to vaccine-related behavior (see Murphy et al., 2021), our results suggest that misinformation may be a factor that could increase mistrust and conspiracy beliefs more than directly influencing vaccine propensity. This point should be investigated in future studies on vaccine propensity addressing the role of mistrust in official information sources in forming paranoid and conspiracy beliefs about such sources.

In the mediation model on vaccine propensity, we showed how MMS mediated the negative effect of distress and paranoia, while BCT mediated the positive effect of anxiety. Again, this result revealed a completely different pattern for conspiracy beliefs and mistrust in connecting psychological variables with vaccine propensity. In particular, the absence of the effect of paranoia on BCT seems to be in contrast to the previous literature, which highlighted a prominent role for paranoia in determining conspiracy beliefs (see Goreis and Voracek, 2019). Instead, our result originally showed how paranoia seems to be implicated with a more general mistrust and suspicious stance on which conspiracy beliefs could eventually be based, but only under certain conditions (as discussed below). In fact, mistrust could be considered a more stable and central symptom of paranoia with respect to ideas of persecution (Bell and O'Driscoll, 2018). Our result originally showed how paranoia seems to be implicated with a more general mistrust and suspicious stance on which conspiracy beliefs could eventually be based, but only under certain conditions (as discussed below).

Death anxiety was the only psychological variable to show both a direct effect on vaccine propensity and an indirect effect through BCT. However, such effects had different signs, that is, the direct sign was positive, i.e., it increased the propensity to get vaccinated, and the mediated one was negative, i.e., it reduced the vaccine propensity by increasing BCT. These dissociated effects seemed to contrast each other, but they can be reconciled. Death anxiety could increase trust in the vaccine as a defense against anguish: In a mortality salience experiment, Farias et al. (2013) showed that thoughts and feelings aroused by thinking about their own death could increase belief in science. However, they reported that such belief in science elicited by mortality salience seems more similar to a form of secular “faith” or religious belief, i.e., it serves to cope with a stressful event such as thinking about death. In this vein, people with high ECQ could see the vaccine as a salvific remediation against COVID-19 without showing a paired, real trust in medicine or science. This is in line with our results, in which death anxiety actually increased mistrust in science. Thus, death anxiety could increase vaccination adherence as a form of mitigation of existential fears and concerns (Pastorino et al., 2021), but also increase belief in conspiracy theories for the very same reasons (van Prooijen, 2020). In fact, these theories could have a protective role against death anxiety (Hornsey et al., 2021). Conspiracy theories, while imaginative, could help in explaining a threat event and thus give a greater sensitivity to a difficult situation than the official explanation (Jutzi et al., 2020). This hypothesis should be tested in further experimental research on the topic, e.g., by comparing the presence of conspiracy beliefs or mentality in two groups of participants, the former exposed to a mortality salience induction and the latter to a control condition.

### Clinical Implications: Relationships Between Conspiracy Theories and Paranoia

From a clinical standpoint, our data suggest that the presence of paranoid ideation is more closely linked to a general attitude of mistrust than to belief in conspiracy theories. Mistrust, however, could be a base on which belief in conspiracy theories can grow, ignited by existential threats (van Prooijen, 2020). In fact, an exaggerated response to threats may be triggered in anxious individuals, who adopt conspiracy theories for their psychological need to feel secure (Green and Douglas, 2018). This suggests that a stable disposition resulting from early childhood experience could lead to belief in conspiracy theories. The stability of such a disposition is further supported by the usual structuring of a monological system of beliefs, i.e., belief in one conspiracy theory leads to beliefs in other conspiracy theories (as reported in Darwin et al., 2011). Thus, in order to treat pathological beliefs in patients, a therapist should first deal with their existential anxiety and with their response to that anxiety. Once such anxiety is relieved, the beliefs in conspiracy theories lose strength and then the pathological paranoid nucleus can be treated. We could conceptualize belief in conspiracy theories as a secondary delirium (Jaspers, 1997), in which the pathological and manifest ideas of reference are based on profound and latent, but explainable, existential causes.

### Limitations and Future Directions

Our study is not free of limitations. First, it implied a cross-sectional design in which causal relations can be only interpreted with caution. In our model, we considered the psychological variables as predictors of the BOC-19 factors, while it is possible that also BCT or MMS affects the level of psychological symptoms circularly (as suggested by Del Vicario et al., 2016). In this respect, future longitudinal and experimental studies will provide a better methodological framework in which to test our hypothesis. We would like to point out that a second data collection is already planned on the same BOC-19 and psychological factors in order to assess how the actual presence and availability of the COVID-19 vaccine has changed the trust in vaccination with respect to the first period of the pandemic spread.

A second important limitation of our study was the use of self-report instruments. In fact, they are more prone to bias with respect to experimental methods. To overcome this limitation, in the future, it would be useful to test our hypotheses by means of experimental manipulation such as mortality salience and mood induction (for a review, see Westermann et al., 1996).

Third, we enrolled our sample through an online form using a convenience sample method. This method implied that our participants were all volunteers and thus motivated to participate, with the possibility of introducing a bias when applying our results to the general population. However, this method was the only one feasible at the time of data collection, when most of the Italian population was in quarantine during the first lockdown period. This method also allowed us to collect data from a more variegated pool rather than just the typical pool of students.

## Conclusions

In conclusion, our work sheds a new light on the complex relationship pattern that links psychological distress and paranoia to mistrust and then to the endorsement of conspiracy theories, by highlighting the role of such factors in predicting vaccine propensity. In our model, we disentangled mistrust from conspiracy factors, by reporting how they relate to specific psychological dimensions. This could help in understanding how to successfully fight such distrust stances, reducing the stigma and the isolation of conspiracy believers while increasing trust in scientific organizations and policymakers during such difficult times. If effective strategies are not identified to help reduce attitudes that undermine the effectiveness of vaccination campaigns, this could have a huge negative impact on the global health situation.

## Data Availability Statement

The raw data supporting the conclusions of this article will be made available by the authors, without undue reservation.

## Ethics Statement

This study was reviewed and approved by Research Ethics and Integrity Committee of CNR, Rome. The participants provided their informed consent to participate in this study.

## Author Contributions

LS designed and executed the study, analyzed the data, and wrote the paper. MV contributed to data analysis and wrote the paper. CG designed and executed the study and revised the paper. GB collaborated to study the design and revised the paper. DP contributed to data analysis and wrote the paper. All authors contributed to the article and approved the submitted version.

## Conflict of Interest

The authors declare that the research was conducted in the absence of any commercial or financial relationships that could be construed as a potential conflict of interest.
